# Supplementary material for: PAX6 Expression Patterns in the Adult Human Limbal Stem Cell Niche
Source: Cells. 2023 Jan 23;12(3):400. doi: 10.3390/cells12030400 (PMC9913671; doi:10.3390/cells12030400)
Supplement: Supplementary file 1 [file cells-12-00400-s001.zip › cells-2164219-supplementary.pdf]

PAX6 expression patterns in the adult human limbal stem cell niche

Naresh Polisetti[1*], Günther Schlunck[1], Thomas Reinhard[1]

[1]Eye Center, Medical Center - Faculty of Medicine, University of Freiburg, Killianstrasse 5, 79106, Freiburg, Germany

Supplementary Figure S1: Double immunostaining of corneoscleral section – the sequence of confocal z-stack image (z1-z14). The arrowhead depicts a non-melanocytic cell overlaid on a melanocytic cell.

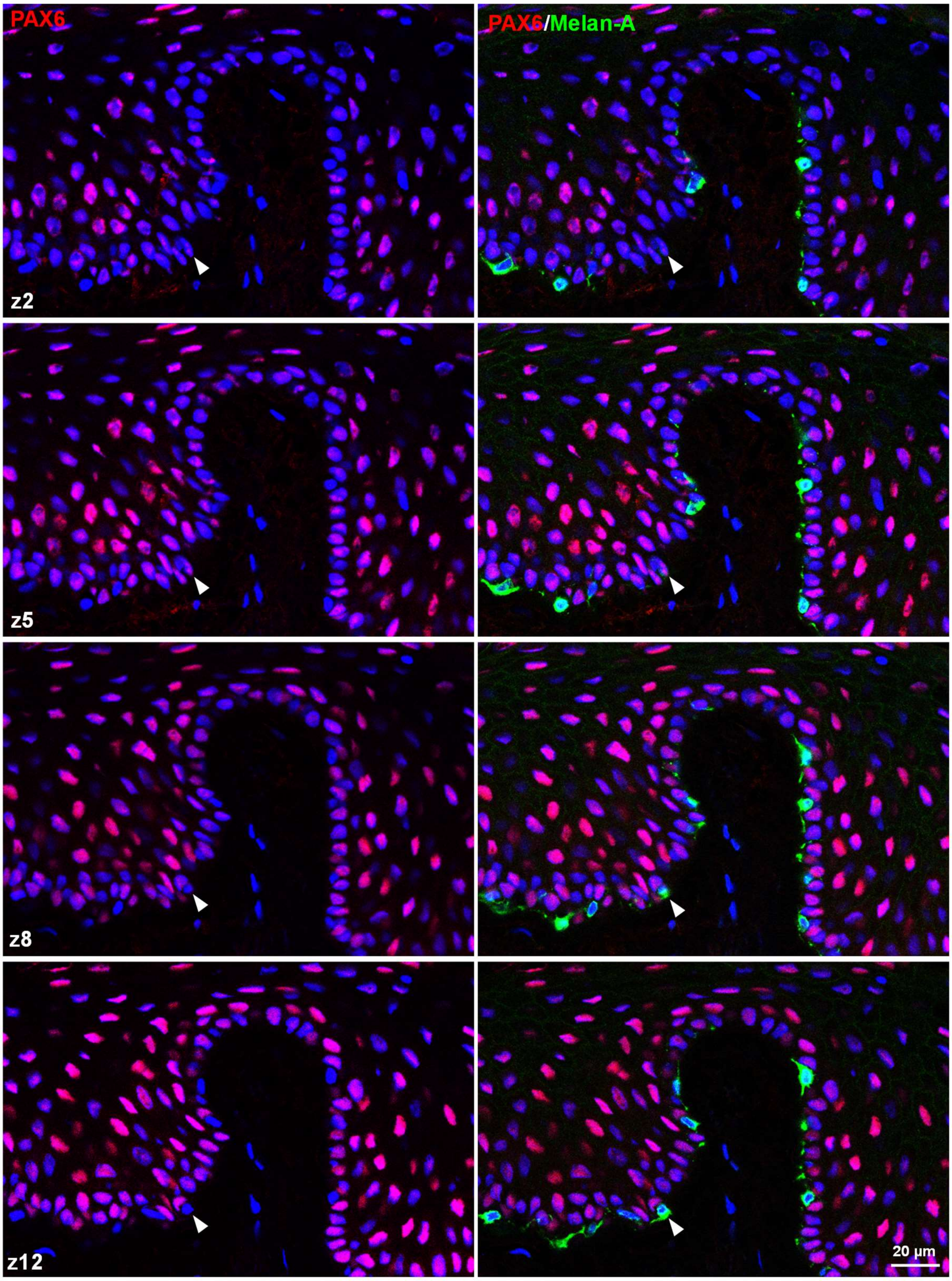

Supplementary Figure S2: Uncropped version of Western blot image shown in Figure 3

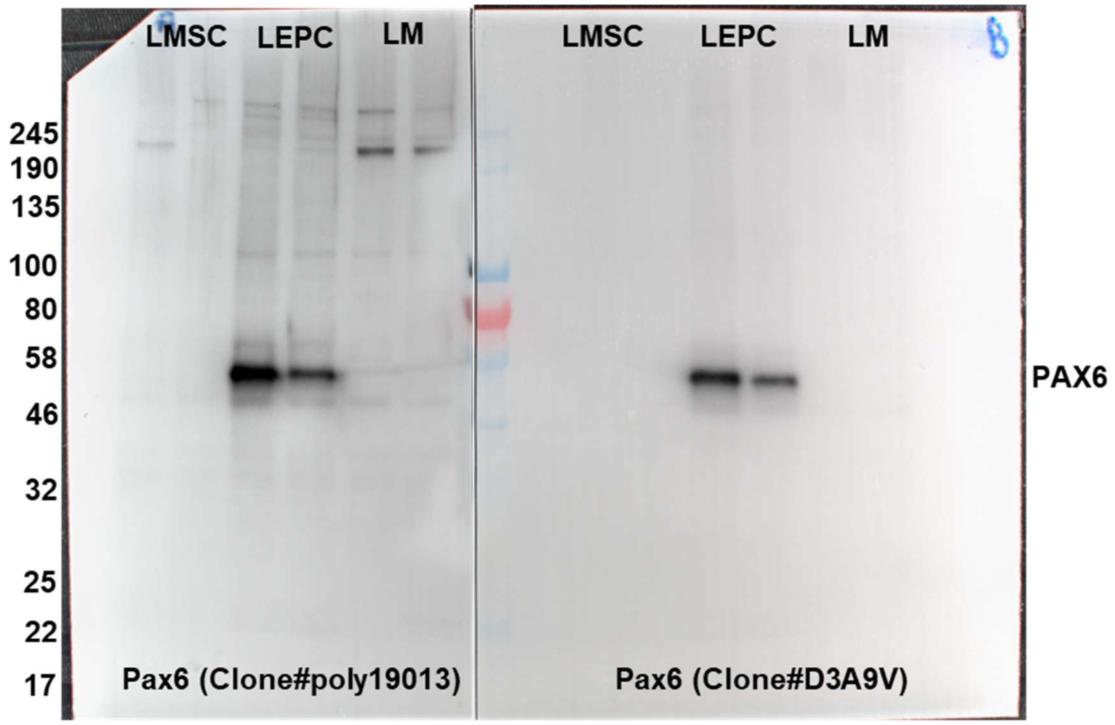

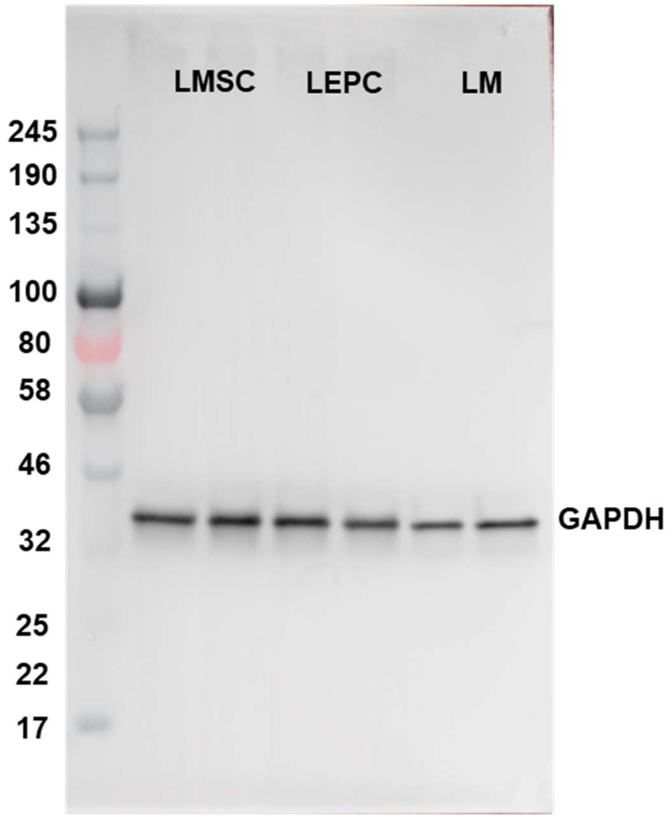

Supplementary Table S1. List of antibodies used

**Antibody (clone), Host species**	**Antibody dilution**	**Application**	**Antibody source**
Cadherin-P/Alexa488, Mouse	5 µl/10^6 cells	Flow cytometry	R&D systems
CD90 APC(5E10), Mouse	5 µl/10^6 cells 1:400	Flow cytometry	BD Biosciences
CD117 PE (A3C6E2), Mouse	5 µl/10^6 cells	Flow cytometry	Miltenyi Biotec
CD117 PE (YB5.B8), Mouse	5 µl/10^6 cells	Flow cytometry	BD Pharmingen
Cytokeratin, pan (AE1/AE3), Mouse	1:100	Immunohistochemistry	DAKO
HMB45 (gp100), Mouse	1:500	Immunohistochemistry	Abcam
IgG2a, k, Isotype PE (MOPC-173), Mouse	5 µl/10^6 cells	Flow cytometry	Biolegend
IgG3, k Isotype FITC (MG3-35), Mouse	5 µl/10^6 cells	Flow cytometry	Biolegend
IgG2a, k Isotype APC (eBM2a), mouse	5 µl/10^6 cells	Flow cytometry	Invitrogen
Melan A, (OTI3E2), Mouse	1:200	Immunohistochemistry	Abcam
PAX6 (D3A9V), Rabbit	1:500<br>1:1000	Immunohistochemistry<br>Westernblotting	Cell Signaling
PAX6 (Poly19013), Rabbit	1:500<br>1:1000	Immunohistochemistry<br>Westernblotting	Biolegend
Vimentin, (280618), Rat	1:500	Immunohistochemistry	Novus Biologicals
